# Endothelial corneal cell damage after pars plana vitrectomy: analogy of different intraocular tamponade agents

**DOI:** 10.22336/rjo.2021.29

**Published:** 2021

**Authors:** Corina Cristina Coman (Cernat), Stella Ioana Patoni (Popescu), Daniel Malița, Simona Stanca, Ovidiu Mușat, Șerban Negru, Horea Feier, Olimpiu Ladislau Karancsi, Cosmin Roșca

**Affiliations:** * Department of Ophthalmology, “Victor Babeș” University of Medicine and Pharmacy, Timișoara, Romania; ** Department of Radiology and Medical Imaging, “Victor Babeș” University of Medicine and Pharmacy, Timișoara, Romania; *** Department of Pediatrics, “Carol Davila” University of Medicine and Pharmacy, Bucharest, Romania; **** Department of Ophthalmology, “Dr. Carol Davila” Central Military University Emergency Hospital, Bucharest, Romania; ***** Department of Oncology, “Victor Babeș” University of Medicine and Pharmacy Timișoara, Romania; ****** Department of Cardiovascular Surgery, “Victor Babeș” University of Medicine and Pharmacy, Timișoara, Romania; ******* Department of Oral Implantology and Prosthetic Restorations on Implants, “Victor Babeș” University of Medicine and Pharmacy, Timișoara, Romania; ******** Department of Ophthalmology, Oculens Clinic, Cluj-Napoca, Romania

**Keywords:** retinal detachment, silicone oil, endotamponade agents, corneal specular microscopy, number of endothelial corneal cells

## Abstract

A reduction in the corneal endothelial cells multitude after anterior pole intervention is well established in numerous researches, but there are few articles that follow the impact of vitreoretinal interventions on the cornea, especially when endotamponade agents are used. The assessment of the endothelial corneal cells is needed since it facilitates the personal evaluation of the functional endothelial stock. Specular microscopy investigation offers a scale of the functional strength of the endothelium of cornea, which is vital before all intraocular interventions.

Endotamponade agents are very suitable and important tools in the surgical treatment of retinal detachment, but their use must be differentiated depending on the uniqueness of each patient. This research shows corneal endothelial damages caused by intraocular tamponade agents of different types in the case of pars plana vitrectomy for cases of multitude retinal detachments. The purpose of the research was to determine the changes that appear in the endothelium of the cornea and to deal with the results when different tamponade agents are used in the surgical cure for retinal detachment. Specular endothelial corneal microscopy records were achieved and the modifications of the following parameters revealed corneal implication: mean endothelial cell densities, average cell area, coefficient of variation, hexagonality and corneal center thickness. On the first day and at three months postoperatively, a statistically significant reduction was observed for the CV, MCD, and HEX parameters (p 0.001), but no statistically significant difference of the two endotamponade agents (for MCD, p=0.15; for CV, p=0.63; for HEX, p=0.93) was noticed. AVG parameter had a statistically significant decrease (p 0.001) and there was also a statistically significant difference of the two endotamponade agents (p=0.03), patients with gas tamponade presenting a superior result. On the first day and at three months postoperatively, the corneal center thickness presented a statistically significant increase (p 0.001) and there was a statistically significant difference between the two endotamponade agents (p=0.03), patients with gas endotamponade presenting a superior result. In conclusion, using the intraocular tamponade agents helps reestablish the functional-anatomical recovery of the retina after surgery, but their special indication must be well-established for each case of retinal detachment.

## Introduction

A short overview of retinal detachment over the past century has shown that progress in this field is not due to chance or accidental disclosure, it represents the work’s results of the ophthalmologist association, and effort still in progress [**1**]. The first who used in vitro air injection, associated with internal diathermy on a retinal rupture was Rosengreen, in 1938 [**2**]. Thus, it provided an internal tamponade role by interrupting the liquid flow currents through the retinal break using gas bubble [**2**]. In 1973, in Miami, Edward Norton updated the internal tamponade with a gas with a longer action than air, expansive: SF6 [**3**]. In 1962, Paul Cibis first used silicone oil without vitrectomy with constant complications [**4**]. Jean High used silicone oil [**5**] on a vitrectomized globe as a prolonged tamponade method, still in use since then. In 1970, Robert Machemer together with biophysicist Jean-Marie Parel developed pars plana vitrectomy with “closed globe” [**6**]; the vitrectomy probe providing cutting, aspiration, and infusion. In 1972, O’Malley perfected vitrectomy by separating the various functions [**7**]. The detach infusion brought a plus in the aspect of the suction cup [**7**]. In another way, endo-lighting provided good quality side lighting and three-y vitrectomy was born [**8**]. Twenty-three-gauge vitrectomy has emerged as a popular surgical technique in recent years [**8**]. The fundamental goods for the surgical modality triumph are the easier access with plane path and with shorter surgical time, smaller conjunctival defects, increased patient convenience and reduced postoperative inflammation [**8**]. Because the cornea is a vital ocular component, being responsible primarily for the dioptric power of the eye, its condition is of major importance in any type of surgery, both preoperative and postoperative [**9**]. Corneal viability is assessed using specular microscopy, which can measure the following parameters related to corneal condition: account of corneal endothelial cells, coefficient of variation, hexagonality, average cell density, and corneal center thickness [**10**-**12**]. The reduction in the endothelial cells corneal number that can sooner or later attend to its decompensation, specifically if it is not healthy, is widely endorsed and validated in numerous researches on surgical interventions aimed at the anterior ocular pole [**13**-**15**]. But then, there is a small number of studies for the effects of posterior pole surgery on the cornea [**16**,**17**], especially when surgery is aided by endotamponades. Silicone gas and oil are widely used as endotamponade methods after retinal detachment surgery [**18**]. Three gases are used with regularity: sulfur hexafluoride, SF6 (reabsorbed in eleven to twelve days); perfluoroethane, C2F6 (reabsorbed in twenty-five to thirty days); perfluoropropane, C3F8 (reabsorbed in about sixty days) [**19**]. C2F6 is not used in the U.S.A, pneumatic retinopexy being most commonly used because the Food and Drug Administration has not approved its use. In Europe, C3F8 is hard to use due to its long period of action, being extra used in analysis. Gas choice confides in its durability and expansion and is reported to the place of the dehiscences [**20**]. Silicone oil is kept intraocular for a duration of partially three months after which it is evacuated according on the condition of the eye’s posterior segment. Silicone oil is commercially available in 5000 and 1000 centistoke forms. 5000 centistoke silicone oil is universally used at present thanks to its fewer aftereffects [**21**].

## Materials and methods

*Ethics permission and patient consent*

This comparative, retrospective, and interventional research admitted 20 eyes of 20 subjects who underwent posterior vitrectomy with gas (20% sulphur hexafluoride (SF6)) or silicone oil (Siluron 1000 centistokes) endotamponade at the Unit of Ophthalmology, “Dr. Carol Davila” Central Military Emergency University Hospital, between March 2019 and April 2020. The study followed the presumption of the Declaration of Helsinki with the International Standard of Good Clinical Practice (ICH-GCP E6 Step 4). Also, the study earned the Local Ethics Committee number 401, endorsed by “Dr. Carol Davila” Central Military Emergency University Hospital Bucharest. After a complete explanation of the examinations, an informed consent was obtained from each subject. In addition, prior to admission, all subjects expressed in writing their informed permission to be exposed to ocular surgery for the treatment of retinal detachment.

*Patients*

A total of 20 patients (13 females, 7 males, aged 54-70) diagnosed with rhegmatogenous or traction retinal detachment, who required posterior vitrectomy aided with endotamponade agents as a treatment solution, from the ophthalmology unit of the “Dr. Carol Davila” Central Military Emergency University Hospital in Bucharest, were involved in the research.

A basic ophthalmologic examination was achieved for each patient, including anterior pole biomicroscopy, indirect and direct fundoscopy, 20/ 20 visual acuity testing, Goldmann tonometry and refractive error determination. Patients who presented any coexisting retinal or corneal sickness, history of eye trauma or any other eye interventions done in the past, including the one for lens replacement, were excluded from the research. Patients who were involved in the study were those with pronounced rhegmatogenous or tractional retinal detachment that required posterior pars plana vitrectomy aided with endotamponade: gas or silicone oil.

*Methods*

Subjects were divided into two groups: group 1 (10 patients: 1 male and 9 females, with 20% SF6 gas endotamponade) and group 2 (10 patients: 6 males and 4 females, with Siluron1000 centistokes endotamponade). Evaluation of the endothelial cell was done on the first postoperative day and on the third month postoperatively, while the gas from the cavity of the vitreous totally disappeared. Silicone oil was required as a longstanding tamponade in all eyes and during the follow-up period it was not extracted. During the study period, no patient had lens opacification removal. After regular eye examinations, the endothelium of the cornea was examined using a specular non-contact microscopy (Nidek Specular Microscope CEM-530) in all patients included in the research. Certified parameters from the system included endothelial cell density (MCD), coefficient of variation of cell size, average cell area (AVG), (CV), hexagonality (HEX) and CT (center corneal thickness). All assessments were measured at the first moment, representing surgery, on the first day and at 3 months postoperatively. As a control mechanism, referral to the other unoperated eye was made.

The same surgeon performed Pars plana posterior vitrectomy under retrobulbar anesthesia in all subjects diagnosed with retinal detachment. The surgical method was the following: later topical disinfection with povidone-iodine, the sterile field and the sterile lid speculum were practiced; insertion of a 23-gauge port through the conjunctiva with infusion and microcannulas at 3.5 mm behind the superotemporal, inferotemporal and superonasal limbus, followed by central and peripheral vitrectomies were done. Next, the removal of the posterior hyaloid face followed, and if mandatory, the inner limiting sheath was extracted after coloring it with triamcinolone. Perfluorocarbon liquid was used if necessary, all the break/ breaks in the retina were placed and circularly bared with laser and after air exchange procedure, gas (SF6,20%) or silicone oil (Siluron® 1000 centistokes) was used to fill the eye, according to the character and location of the retinal detachment. 

For all the operated eyes, the same equal balanced salt solution for ophthalmic use (BSS Plus,Alcon, USA) was utilized as the irrigating intraocular solution. Also, drops of salt solution were practiced to the surface of the eye every 20–40 seconds to avoid damage of the corneal epithelium. Antibiotic and steroid drops for the eye were administered to all patients five times daily, for over 1 month after surgery.

We performed bilateral endothelial corneal microscopy in all patients preoperatively, on the first day and three months postoperatively, for counting endothelial cells, coefficient of variation in cell size, central cell area, hexagonality and corneal center thickness. 

Patients were subsequently released after verification of retinal attachment and then reexamined after three months, using endothelial corneal microscopy; the results were noted.

All parameters of the studied values were determined using similar working manners. For the analyzing and systematization of the info, the Excel program of Microsoft Office 365 suite was used. The graphical images, as well as the data statistical analysis, were achieved with the same program, together with “add-ins”, such as WinStat and XL-stat. Online support was provided by Prof. Richard Lowry-Vassar College Poughkeepsie, NY USA, through the link www.vassarstats.netwas used for the statistical significance calculation of the recorded results.

*Statistical analysis*

To obtain reports between various values of the analyzed parameters, the average values were determined, and standard deviation and the Student test (t-test) was calculated, its statistical significance being represented by the p values. Statistical significance of all the results was interpreted reported to the value of the p coefficient: the results were not statistically significant if p value was greater than 0.05; for values of p between 0.05 and 0.001, the results were treated as highly statistically significant, whereas the p values below 0.001 were considered very highly statistically significant.

## Results

*Patients*

**Fig. 1:**
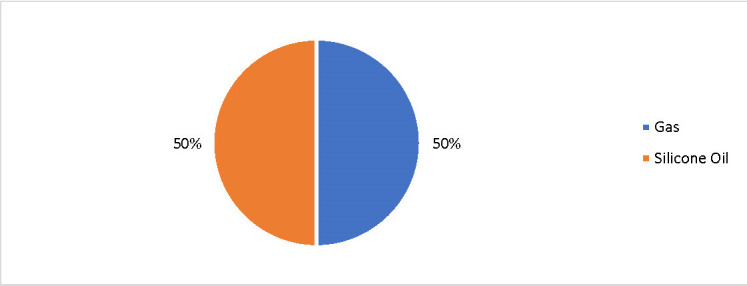
Patients’ distribution based on the endotamponade agents used

All subjects’ dissemination reporting to different endotamponade agents used is presented in **Fig. 1**.

**Fig. 2:**
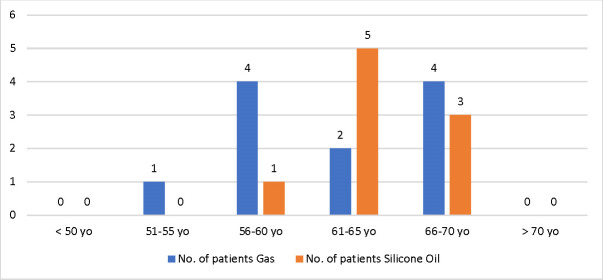
Patients’ distribution based on their age and on the endotamponade agents

Patients’ distribution according to their age and endotamponade agents is shown in **Fig. 2**.

*Varied parameters*

There is a linear reduction for the parameters MCD, AVG, CV, HEX in all patients included in the study regardless of the intraocular tamponade agent used during surgery, both immediately and at 3 months postoperatively. For the CT parameter, a slight merger was observed both immediately and at 3 months postoperatively for all patients disregarding the endotamponade agent used. 

**Fig. 3:**
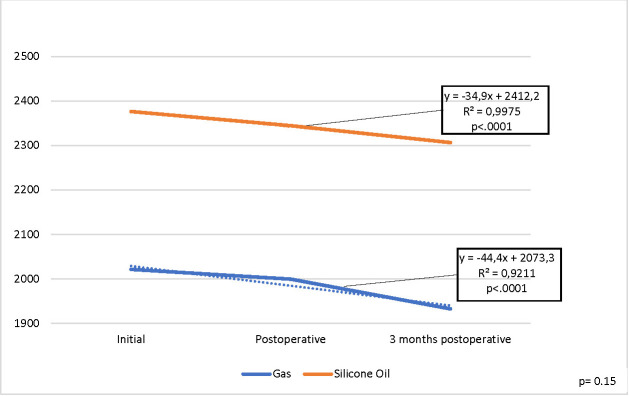
Evolution of mean MCD values for the operated eye

A statistically significant decrease was observed immediately and at 3 months postoperatively for the MCD parameter (p 0.001), as presented in **Fig. 3**, but there was no statistically significant difference (p=0.15) between the two endotamponade agents.

CV values lowered postoperatively for all patients, being statistically significant (p .0001), but there was no statistically significant difference (p=0.63) between the two endotamponade agents.

**Fig. 4:**
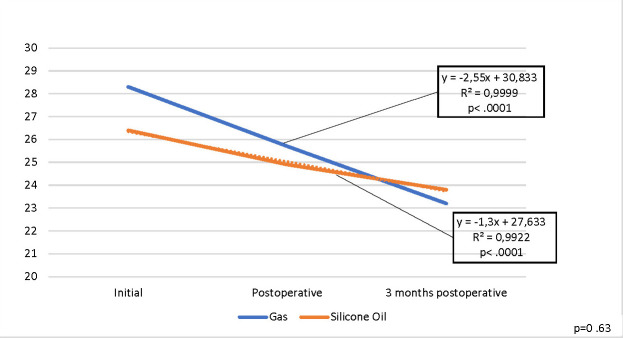
Evolution of mean CV values for the operated eye

The results are presented in **Fig. 4**.

**Fig. 5:**
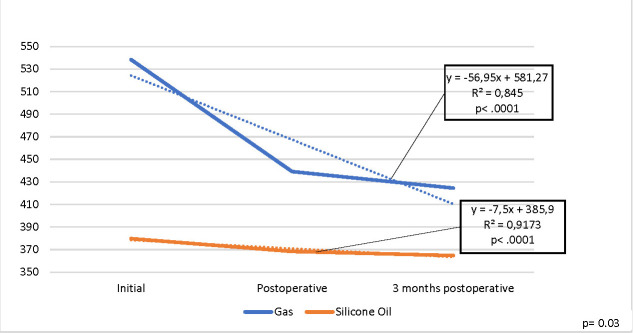
Evolution of mean AVG values for the operated eye

In all patients, AVG values decreased postoperatively, being statistically significant (p .0001) and a statistically significant difference (p=0.03) was observed between the two endotamponade agents; patients with gas endotamponade had an exceeded result presented in **Fig. 5**.

**Fig. 6:**
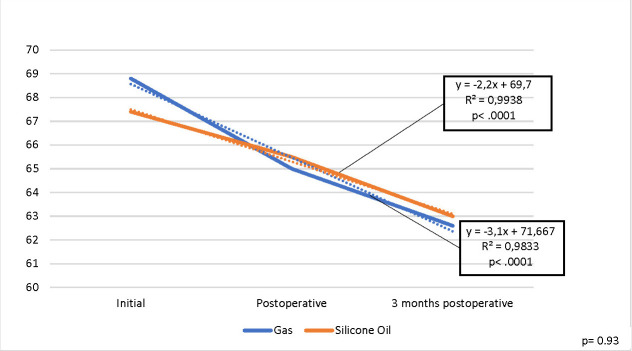
Evolution of mean HEX values for the operated eye

HEX values decreased for all patients (p .0001), but there was no statistically significant difference between the two endotamponade agents (p=0 .93) (**Fig. 6**).

**Fig. 7:**
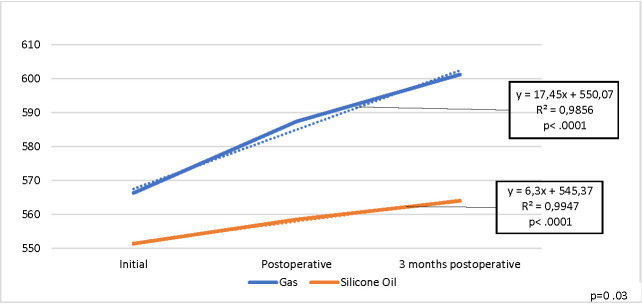
Evolution of mean CT values for the operated eye

Statistically significant increased CT values were noticed postoperatively for all patients (p .0001), there was a statistically significant difference between the two endotamponade agents (p= 0.03) and gas endotamponades had a better result (**Fig. 7**).

*Analogy of values of the analyzed parameters according to the endotamponade agent*

An analogy between the average values of a particular analyzed parameter according to the utilized endotamponade agent was also made. No statistical differences could be detected for the first day postoperative or at three months postoperatively analysis. The only significant statistical difference observed was between the basic measurements for AVG of the eyes that underwent surgery (the surface was significantly bigger when gas endotamponade was utilized) (**Table 1**).

**Tabel 1:** Analogy between average values for each analyzed parameter, depending on the endotamponade agent utilized

Average Values	Initial			Postoperative			3 months postoperative		
	Gas	Silicone Oil	*P*	Gas	Silicone Oil	*P*	Gas	Silicone Oil	*P*
MCD operated eye (cells/ sqmm)	2021.4	2376.3	0.15	1999.5	2344.4	0.16	1932.6	2306.5	0.15
CV operated eye (%)	28.3	26.4	0.48	25.7	24.9	0.77	23.2	23.8	0.81
AVG operated eye (sqμm)	538.4	379.7	0.01	439.2	368.3	0.17	424.5	364.7	0.27
HEX operated eye (%)	68.8	67.4	0.65	65	65.5	0.85	62.6	63	0.88
CT operated eye (μm)	566.3	551.4	0.54	587.4	558.5	0.27	601.2	564	0.15

## Discussion

Gas and silicone tamponade has played a broad significant role in the retinal detachment treatment once Rosengren [**22**] described the use of air for tamponade retinal breaks and Norton [**23**] described the use of sulphur hexafluoride in the extension of the intraocular gas tamponade. Zivojnovic et al. [**24**] encouraged the use of long-lasting intraocular tamponades in closing iatrogenic retinal holes or retinotomies created during vitreoretinal surgery. In addition, vitreous surgery techniques have encouraged the use of endotamponade agents such as silicone oil and gas to allow reapplication of the retina to the retinal pigment epithelium during surgery [**25**]. Despite the important role of intraocular tamponade agents in posterior pole surgery, they can cause endothelial corneal impairment, which auxiliary leads to reduced visual acuity [**26**]. The aim of this research was to find the repercussions of endotamponade agents on the endothelium of the cornea.

In this research, we demonstrated a substantial decrease in the number of endothelial corneal cells by following the parameters of specular endothelial microscopy: MCD, AVG, CV and HEX. No important complications were noted during the follow-up of the analysis due to the type of endotamponade agent used, except for a slight inflammation of the anterior pole that was present in all patients operated on almost regularly.

In 2015, E. Cinar and collaborators [**27**], who studied three groups, showed that MCD, AVG, CV and HEX values were lower in all patient groups, but no difference was noticed between the group with gas endotamponade compared to the one in which silicone oil was used. In our study, all the values had a statistically significant decrease, and for the AVG parameter we observed a statistically significant difference between the two endotamponade agents (p= 0.03), patients with gas endotamponade having a better result.

Pars plana vitrectomy and the use of different endotamponade agents lead to corneal endothelial cell modifications expressed by loss of endothelial cell pleomorphism [**28**]. Using different types of endotamponade agents could be a dangerous factor for increasing endothelial cell loss after posterior vitrectomy [**29**].

In this paper, we established a statistically significant endothelial cells loss after vitreoretinal surgery in all groups, both immediately and at 3 months postoperatively, proved by changes of MCD, AVG, CV and HEX. Endotamponade intraocular agents are widely used for cure of complex cases of retinal detachment.

Regular patients monitoring is essential, especially when prolonged tamponade is required. All the reduced values were statistically significant. For MCD and CD it was statistically significant both immediately (p 0.001) and at 3 months postoperatively (p 0.001), but no statistically significant difference was observed between the two intraocular tamponade agents, for MCD p=0.15 and for CV p=0.63. For AVG and HEX, the decrease was also statistically significant both immediately (p 0.001) and at 3 months postoperatively (p 0.001). We also established a statistically significant difference between the two endotamponade agents, for AVG p=0.03 but not for HEX p=0.93. However, the study had several limitations. 

The first one was the relatively reduced number of patients who participated in the research.

Also, the time of the surgery was not set, knowing that this is also an important factor that causes a decrease in the amount of corneal endothelial cells. Another limitation was that just a short-acting gas endotamponade (SF6) and one kind of silicone oil (Siluron 1000) were used as endotamponade agents. 

## Conclusion

To conclude, posterior vitrectomy adjusted with gas or silicone oil intraocular tamponade result into a decrease in the amount of corneal endothelial cells. Several additional factors affect the decrease in the amount of corneal endothelial cells during intervention, among which the following should be mentioned: phototoxicity, temperature changes and fluid turbulence.

In addition, particular researches are needed to demonstrate the consequence of vitreoretinal surgery regarding the corneal endothelium.

**Conflict of Interest statement**

The authors state no conflict of interest.

**Informed Consent and Human and Animal Rights statement**

Informed consent has been obtained from all individuals included in this study.

**Authorization for the use of human subjects**

This study was granted by the Local Ethics Committee of “Dr. Carol Davila” Central Military Emergency University Hospital, Bucharest (number 401) and was conducted in accordance with the Declaration of Helsinki and with the International Standard of Good Clinical Practice (ICHGCP E6 Step 4). All subjects expressed their informed consent in writing.

**Acknowledgments**

Corina Cristina Coman (Cernat), Daniel Malița and Simona Stanca had equal contribution to the paper.

Professional editing, linguistic and technical assistance was performed by Irina Radu, Individual Service Provider, certified translator in Medicine and Pharmacy (certificate credentials: series E no. 0048).

**Sources of Funding**

No funding was received.

**Disclosures**

None.

**Availability of data and materials**

All data and materials supporting the issues of the present study are disposable in the published article.

**Authors’ contributions**

CCC designed the study and was liable for the acquirement, and reading of the data above. SN provided scientific advice. OM, SIP, HF, CR were involved in the design of the project, review of the outcome, and revised the manuscript. DM, SS, SN, OLK were involved in the outlook and drafting of the paper and revised the manuscript. All authors read and authorized the conclusive manuscript.

**Competing concern**

The authors state that they have no competing concerns.
